# Design and Characterization of Ethosomes for Transdermal Delivery of Caffeic Acid

**DOI:** 10.3390/pharmaceutics12080740

**Published:** 2020-08-06

**Authors:** Supandeep Singh Hallan, Maddalena Sguizzato, Paolo Mariani, Rita Cortesi, Nicolas Huang, Fanny Simelière, Nicola Marchetti, Markus Drechsler, Tautgirdas Ruzgas, Elisabetta Esposito

**Affiliations:** 1 Department of Chemical and Pharmaceutical Sciences, University of Ferrara, I-44121 Ferrara, Italy; hllsnd@unife.it (S.S.H.); maddalena.sguizzato@unife.it (M.S.); mrcncl@unife.it (N.M.); 2 Biofilms—Research Center for Biointerfaces, Faculty of Health and Society, Malmö University, SE-20506 Malmö, Sweden; 3 Department of Life and Environmental Sciences, Polytechnic University of Marche, I-60131 Ancona, Italy; p.mariani@staff.univpm.it; 4 CNRS, Institut Galien Paris-Saclay, Université Paris-Saclay, 92296 Châtenay-Malabry, France; nicolas.huang@universite-paris-saclay.fr (N.H.); fanny.simeliere@universite-paris-saclay.fr (F.S.); 5 Bavarian Polymer Institute (BPI) Keylab “Electron and Optical Microscopy” University of Bayreuth, D-95440 Bayreuth, Germany; markus.drechsler@uni-bayreuth.de

**Keywords:** ethosome, caffeic acid, penetration enhancers, in vitro diffusion, oxygen electrode

## Abstract

The present investigation describes a formulative study aimed at designing ethosomes for caffeic acid transdermal administration. Since caffeic acid is characterized by antioxidant potential but also high instability, its encapsulation appears to be an interesting strategy. Ethosomes were produced by adding water into a phosphatidylcholine ethanol solution under magnetic stirring. Size distribution and morphology of ethosome were investigated by photon correlation spectroscopy, small-angle X-ray spectroscopy, and cryogenic transmission electron microscopy, while the entrapment capacity of caffeic acid was evaluated by high-performance liquid chromatography. Caffeic acid stability in ethosome was compared to the stability of the molecule in water, determined by mass spectrometry. Ethosome dispersion was thickened by poloxamer 407, obtaining an ethosomal gel that was characterized for rheological behavior and deformability. Caffeic acid diffusion kinetics were determined by Franz cells, while its penetration through skin, as well as its antioxidant activity, were evaluated using a porcine skin membrane–covered biosensor based on oxygen electrode. Ethosome mean diameter was ≈200 nm and almost stable within three months. The entrapment of caffeic acid in ethosome dramatically prolonged drug stability with respect to the aqueous solution, being 77% *w*/*w* in ethosome after six months, while in water, an almost complete degradation occurred within one month. The addition of poloxamer slightly modified vesicle structure and size, while it decreased the vesicle deformability. Caffeic acid diffusion coefficients from ethosome and ethosome gel were, respectively, 137- and 33-fold lower with respect to the aqueous solution. At last, the caffeic acid permeation and antioxidant power of ethosome were more intense with respect to the simple solution.

## 1. Introduction

Human skin can be affected by a broad range of disorders, spanning from inflammatory diseases to skin cancer, many of which did not find an efficacious therapy; thus, there is an unmet need for effective strategies to treat dermatological pathologies [1,2,3,4]. Many antioxidant compounds possess therapeutic activities—for instance, caffeic acid (CA) is a naturally occurring hydroxycinnamic acid with remarkable antioxidant, anti-inflammatory, and antiproliferative properties [5,6,7,8]. Notably, some studies have demonstrated CA’s potential in the treatment of skin inflammatory pathologies, such as psoriasis. Moreover, thanks to its antioxidative power, CA can exert photoprotective action towards DNA damage, protecting skin against aging and preventing melanoma [9,10,11,12]. Despite the pharmaceutical potential of antioxidant molecules, some of them are prone to degradation, being sensitive to oxygen, light, and high temperature [13]. In addition, it should be noted that the barrier effect of *stratum corneum* often represents an obstacle to active molecules permeation, hampering their final pharmacological effect. In this respect, antioxidant encapsulation could represent an appropriate strategy to control chemical instability and to improve their performances under skin application [14]. Among the different micro- and nano-systems provided for drug encapsulation and transdermal delivery, ethosomes (ETHOs) represent a smart strategy. Indeed, ETHOs are vesicular systems constituted of phospholipids, such as phosphatidyl choline (PC), ethanol (20–45%), and water [15,16,17,18,19]. The multilamellar vesicles of ETHOs allow to improve loading of poor soluble molecules with respect to the well-known liposomes. Indeed, the presence of ethanol stabilizes the vesicles and controls their entrapment efficacy [15,16]. Moreover, many studies have demonstrated the ETHO capability to achieve transdermal delivery of drugs due to a synergy between ethanol and phospholipids. In fact, on one hand, ethanol renders vesicles softer with respect to liposome, and on the other ethanol and phospholipids enhance drug penetration. Indeed, ethanol is able to disorganize the *stratum corneum* barrier, opening spaces for ETHO crossing, while the peculiar structure of phospholipids, similar to skin lipids, promotes ETHO permeation [16,17,18]. On this matter, some authors have demonstrated the presence of intact vesicles in the skin strata, suggesting that ETHOs can overcome the *stratum corneum,* allowing deep drug permeation [20,21,22]. In this context, the object of the present investigation is a pre-formulative study aimed at the development of ETHOs for CA solubilization and delivery through the skin. Particularly, the ETHO capability to control CA degradation has been investigated. Moreover, since low viscous vehicles need a thickening agent to obtain the appropriate residence time when applied on the skin, the copolymer poloxamer 407 has been added to ETHOs. This copolymer, constituted of polyoxyethylene-polyoxypropilene units, possesses thermogelling properties when dispersed in water, passing from fluid to semi-solid materials over a transition temperature T_sol-gel_ [23,24]. The structural organization and mechanical properties of ETHO formulations have been investigated by small-angle X-ray scattering (SAXS) and rheometric analyses. Franz cell has been employed to detect the influence of ETHO entrapment on CA diffusion kinetics. Finally, an amperometric study has been conducted in order to evaluate CA permeation through the skin. Particularly, an in vitro tool based on skin covered oxygen electrode (SCOE), has been exploited to assess the kinetics of CA penetration through *stratum corneum* and its involvement in antioxidative reactions in skin at the presence of hydrogen peroxide, H_2_O_2_ [25]. In the SCOE apparatus, an electrode allows registration of O_2_ concentration changes in the membrane due to reactions of H_2_O_2_ and polyphenols in the skin [26]. This electrochemical monitoring enables to obtain a reliable prediction of in-vivo cutaneous antioxidant administration [27].

## 2. Materials and Methods

### 2.1. Materials

Caffeic acid (CA), sodium citrate dihydrate, citric acid monohydrate, sodium chloride, 2,2-Diphenyl-1-picrylhydrazyl (DPPH), dimethyl sulfoxide (DMSO), and ascorbic acid were purchased from Sigma-Aldrich (St. Louis, MO, USA). The soybean lecithin (PC) (90% phosphatidylcholine) used for ETHO preparation was Epikuron 200 from Lucas Meyer (Hamburg, Germany). The copolymers poly(ethylene glycol)-block-poly(propylene glycol)-block-poly(ethylene glycol) Pluronic F127 (poloxamer 407, p407) (PEO98-POP67-PEO98) was obtained from BASF (Ludwigshafen, Germany). Nylon and polycarbonate membranes (pore size 200 and 50 nm respectively) were purchased from Merck (Milan, Italy). The Clark-type oxygen electrode was purchased from UAB “OPTRONIKA” (Vilnius, Lithuania). All solutions were prepared in water purified by the Milli-Q system (Merck Millipore, Billerica, MA, USA) with resistivity of 18.2 Ω cm. Solvents were of HPLC grade, and all other chemicals were of analytical grade.

### 2.2. Caffeic Acid Solubility and Stability Evaluation

Solubility of CA was determined by saturating water, or ethanol/water 30:70 solution, with an excess of drug in sealed glass vials, at 22 °C. The obtained saturated solutions were horizontally shaken at 300 rpm for 8 h, in the dark and centrifuged at 3000× *g* for 15 min. The supernatant was withdrawn and filtered through a regenerate cellulose filter membrane, 0.22 μm pore size, 25 mm diameter (Millipore-Sigma-Aldrich Merck, Darmstadt, Germany). CA concentration was determined by mass spectrometry analysis as below reported. In order to detect CA chemical stability, 1 mL samples of CA aqueous solution were stored in different conditions, namely at 4, 22, and 40 °C for 30 days. The CA concentration was determined weekly by a LC-MS/MS system (Thermo Scientific, Waltham, MA, USA) composed of a micro HPLC Surveyor Plus (pump, column, thermostated compartment, autosampler, and solvent delivery system) and an LTQ XL mass spectrometer according to a previously developed method [28]. CA was separated by HPLC under gradient elution condition with H_2_O + 0.1% *v/v* formic acid and acetonitrile + 0.1% *v/v* formic acid as mobile phases. Flow rate was 150 μL/min and gradient was 5–30% acetonitrile in 20 min. Chromatographic column was a Symmetry (Waters) 100 × 2.1 mm, packed with 3 μm fully porous particles and thermostated at 25 °C. Main MS conditions were as following: capillary temperature 275 °C; sheat gas 40 au; source voltage—4.00 kV; capillary voltage—6.00 V; tube lens—47.20 V. Detection of CA was performed by selected reaction monitoring (SRM) mode. The SRM detection was operated in the negative electrospray ionization mode using the transitions *m*/*z* 179 ([M-H]^–^) → 135.

Stability profiles were evaluated by mathematical modelling, particularly the following mathematic models were applied to the percentage of residual drug as follows [29]:

Zero order: *Q_t_* = *Q*_0_ + *Kt* (1)

First order: ln*Q_t_* = ln*Q*_0_ − *Kt* (2)

where *Q_t_* (%) is the percentage of residual drug at time *t*, *Q*_0_ is the initial value of *Q_t_*, *t* is the time, and *K* is the coefficient corresponding to zero (1) or first (2) order kinetic models.

### 2.3. Ethosome Preparation

ETHO preparation was simply obtained by dropping bidistilled water into an ethanolic solution of PC (30% *w*/*v*) [22]. Namely, water was slowly added to the ethanol phase up to a final 70:30 (*v*/*v*) ratio, magnetically stirring the system at 750 rpm (IKA RCT basic, IKA^®^-Werke GmbH & Co. KG, Staufen, Germany) for 30 min at room temperature in the dark. In the case of CA containing ETHO (CA-ETHO), the drug (1 mg/mL) was added to PC ethanol solution before the addition of water. Table 1 reports the ETHO compositions. After ETHO preparation, the ethanol presence was checked by gas-chromatographic analysis (Agilent G4407) equipped with a thermal conductivity detector and a Restek Porapak Q porous polymers packed column (80/100 mesh, 20 m). The carrier gas flow rate was set at 200 kPa, the injector temperature was 200 °C, the column temperature was 150 °C, and the detector temperature was 180 °C. One microliter of 1:10 diluted ETHO sample (*n* = 3) was injected and ethanol (retention time around 3.3 min) was determined by external calibration [30].

### 2.4. CA Content of Ethosomes and Encapsulation Efficiency Evaluation

To determine the total amount of drug in the formulations, CA content was quantified by CA-ETHO dilution with ethanol (1:10, *v*/*v*) followed by magnetic stirring for 30 min [15,22]. After filtration by nylon syringe filters (0.22 μm pores), the total content of CA (*T*_CA_) was analyzed by HPLC, as reported below.

To determine the amount of drug associated to vesicles, the encapsulation efficiency (EE) of CA in ETHO has been determined by ultracentrifugation. Five hundred microliters of CA-ETHO were poured in a centrifugal filter (Microcon centrifugal filter unit YM-10 membrane, NMWCO 10 kDa, Sigma-Aldrich, St. Louis, MO, USA) constituted of two vials—the upper to recover retentate, and the other to collect filtrate. Under ultra-centrifugation (Spectrafuge™ 24D Digital Microcentrifuge, Woodbridge, NJ, USA) at 8000 rpm—the upper vial retained ETHO-encapsulating drug, while the lower contained the free drug. After 20 min, a 100 μL aliquot withdrawn from the upper vial has been diluted, stirred, filtered as above reported and analyzed by HPLC. The EE was determined as follows:

*EE* = *CA*/*T*_CA_ × 100 (3)

where *CA* is the amount of drug retained in ETHOs and *T*_CA_ is the total content of CA.

### 2.5. Cryo-Transmission Electron Microscopy (Cryo-TEM)

In order to vitrify samples for cryo-transmission electron microscopy, sample droplets (2 μL) were put for some seconds on a lacey carbon filmed copper grid (Science Services, München, Germany) [31]. Afterward, most of the liquid has been removed by a blotting paper, obtaining a thin film stretched over the lace holes. The rapid immersion of specimen into liquid ethane cooled to approximately 90 K (−180 °C) by liquid nitrogen in a temperature-controlled freezing unit (Leica EMGP, Leica, Germany) instantly allowed their vitrification. All sample preparation steps were conducted at a controlled constant temperature in the Leica EMGP chamber. The vitrified specimen was transferred to a Zeiss/Leo EM922 Omega EFTEM (Zeiss Microscopy GmbH, Jena, Germany) transmission electron microscope using a cryoholder (CT3500, Gatan, Munich, Germany). During the microscopy observations, sample temperature was kept below 100 K. Specimens were examined with reduced doses ≈1000–2000 e/nm^2^ at 200 kV. Zero-loss filtered images (Δ*E* = 0 eV) have been recorded by a CCD digital camera (Ultrascan 1000, Gatan, Munich, Germany) and analyzed using a GMS 1.9 software (Gatan, Munich, Germany).

### 2.6. Photon Correlation Spectroscopy (PCS)

Vesicle size analysis of ETHO, CA-ETHO, ETHO-pol, and CA-ETHO-pol was conducted using a Zetasizer Nano-S90 (Malvern Instr., Malvern, UK) with a 5 mW helium neon laser and a wavelength output of 633 nm. Measurements were performed at 25 °C at a 90° angle and a run time of at least 180 s. Samples have been diluted with bi-distilled water in a 1:20 *v/v* ratio. Data were analyzed using the “CONTIN” method [32]. Measurements were performed thrice for three months from ETHO production.

### 2.7. X-Ray Scattering

Small-angle X-ray scattering (SAXS) experiments were performed at the bioSAXS beamline B21 of Diamond Light Source (Harwell, UK). ETHO, CA-ETHO, pol, CA-pol, and CA-ETHO-pol samples were placed into PCR tubes in an automated sample changer and then transferred to a temperature-controlled quartz capillary and exposed for 1 s, acquiring 30 frames at 35 °C in order to check equilibrium conditions and to eventually monitor radiation damage. Data collection was performed using a Pilatus Dectris 2 M detector. The X-ray wavelength λ was 0.10 nm and the explored *Q*-range (*Q* is the modulus of the scattering vector, defined as 4π sin θ/λ, where 2θ is the scattering angle) was 0.1 to 4.4 nm^−1^. Two-dimensional data were corrected for background, detector efficiency and sample transmission and then radially averaged to derive I(*Q*) vs. *Q* curves [33].

### 2.8. Antioxidant Activity

The antioxidant activity of CA-ETHO was evaluated by the free radical scavenging activity DPPH and photochemiluminescence methods [34,35,36].

The effect of CA on DPPH• radical was estimated according to recommendations of Marinova and Batchvarov with some modifications [34]. All solutions were prepared in ethanol. The stock DPPH• solution was prepared by dissolving 7 mg DPPH in 10 mL ethanol and then stored until needed. The control solution was obtained by mixing the stock solution with ethanol (1:10, *v*/*v*) to obtain an absorbance of approximately 1.0 unit at 517 nm. A number of CA-ETHO dilutions in ethanol (ranging from 1:10 to 1:100, *v*/*v*) was prepared in ethanol (total volume 225 µL), mixed with the same amount (25 µL) of DPPH solution in ethanol and incubated at 400 rpm for 30 min in the dark, at 21 °C. Ethanol was used as the blank control. The absorbance decreasing was recorded at 517 nm. For all evaluated assays, absorbance measurements were performed in triplicate in a microplate reader. The radical scavenging activity was expressed in terms of % inhibition of DPPH absorbance:

DPPH scavenging activity = (A control − A sample)/A control × 100%(4)

where A control is the absorbance of the control (DPPH solution) and A sample is the absorbance of the sample (DPPH solution plus CA-ETHO). Different CA solution concentrations were evaluated to determinate the ability to scavenge DPPH• radicals by the 50% signal inhibition concentration (IC50 values express the sample concentration required to scavenge 50% of the DPPH-free radicals) in ascorbic acid equivalents and using linear regression analysis. For ascorbic acid the IC50 value is roughly 6.1 μg/mL. Absorbance have been measured with Safire plate reader (Tecan Trading AG, Männedorf, Switzerland) [35]. In addition, the total antioxidant value of CA-ETHO and CA ethanol solution was measured by photochemiluminescence assay using Photochem, ACL kit (Analytica Jena AG, Jena, Germany). The antioxidant values of the samples were determined following the manufacturer instructions, and the results are expressed in terms of trolox equivalents (TE) [36].

### 2.9. Evaluation of Ethosome Stability

To check ETHO size stability, PCS measurements of samples stored in the light at 22 °C were performed thrice after 7, 15, 30, 60, and 90 days from ETHO production.

Chemical stability has been determined by analyzing residual CA content in ETHO once a week for 30 days and then monthly, up to six months from production, with the method above reported (Section 2.4). Residual CA content has been expressed as percentage of T_CA_ evaluated after CA-ETHO preparation. As control, CA content in a CA ethanol/water solution (30/70, *v*/*v*), stored at 22 °C, was measured.

### 2.10. Gel Preparation

P407 gel (pol) was prepared by slow addition of the polymer into cold water (5–10 °C) under magnetic stirring (750 rpm) up to a 15% *w*/*w* final concentration (Table 1). The container was sealed and left in a refrigerator at 5 °C overnight. In case of CA-pol, the drug was solubilized in the pre-formed gel under swirling agitation at 30 Hz frequency. The maximum CA concentration was 0.5 mg/mL [37,38].

Ethosome gels were obtained by direct addition of p407 (15% *w*/*w*) into pre-formed ETHO. Namely, the polymer has been gradually added under magnetic stirring (750 rpm) at 5 °C for 3 h. CA final concentration was 0.85 mg/mL. Table 1 reports the compositions of ETHO-pol and CA-ETHO-pol.

### 2.11. Rheological Measurements

Rheological measurements were performed with an AR-G2 controlled-stress rotational rheometer (TA Instruments, New Castle, DE, USA) [37]. The geometry used was an aluminum cone-plate with a diameter of 40 mm, an angle of 1° and a truncation of 28 µm, equipped with a solvent trap in order to prevent solvent evaporation during the experiments. The viscoelastic properties of the gels (elastic modulus G’ and viscous modulus G’’) were assessed in oscillation mode. Oscillation frequency was set at 1 Hz and the deformations applied were all in the linear regime. Temperature ramps from 5 to 50 °C were obtained at a temperature rate of 1 °C/min and were controlled by a Peltier plate. Before starting the experiments, a 2-min conditioning time at 5 °C was applied. Measurements were performed thrice at least for each sample, to ensure reproducibility.

### 2.12. Deformability Measurement

The deformability of ethosomal vesicles was determined by an extrusion method [39]. Ethosomal dispersions (ETHO, CA-ETHO) and ethosome gels (ETHO-pol and CA-ETHO-pol) were extruded through polycarbonate filter membrane (pore diameter 50 nm) using a stainless steel, 25-mm diameter filter holder (extruder, Lipex Biomembranes, Vancouver, BC, Canada), applying 2.5 bars of pressure at 25 °C. The volume of ETHO formulation extruded in 1 (ethosome dispersions) or 25 (ethosome gels) min was measured. Vesicles size has been measured by PCS before and after the extrusion. The deformability of vesicles membrane was calculated according to the following equation:

*Def* = *J* × (*r*v/*r*p)^2^ (5)

where *Def* is the vesicle deformability; *J* is the ratio between the volume of extruded formulation (mL) and the time of extrusion (min); *r*v is the vesicle size (after extrusion); and *r*p is the pore size of the filter membrane.

### 2.13. In Vitro Diffusion Experiments

In vitro CA diffusion has been evaluated by Franz cells. Particularly, nylon membranes (2 cm diameter, pore size 0.2 μm) were hydrated in distilled water for 1 h before being placed in Franz-type diffusion cells produced by Vetrotecnica (Padova, Italy) [24,37]. The exposed membrane surface area was 0.78 cm^2^ (1 cm diameter orifice). Franz cells were constituted of a lower receptor compartment and an upper donor compartment sealed to avoid evaporation during the experiments. Five milliliters of bidistilled water have been poured in the lower section, stirred at 500 rpm by a magnetic bar, and thermostated at 32 ± 1 °C during all the experiments [24,40]. Approximately 1 g of CA-sol (CA 0.5 mg/mL), CA-pol (CA 0.5 mg/mL), CA-ETHO (CA 1 mg/mL), or CA-ETHO-pol (CA 0.85 mg/mL) was placed in the donor compartment on the membrane surface. Two hundred microliters of receptor phase were withdrawn at predetermined time intervals (1–5 h) and analyzed for CA content using HPLC. Each removed sample was replaced with an equal volume of simple receptor phase. The CA concentrations were determined six times in independent experiments and the mean values ± standard deviations were calculated. The mean values were then plotted as a function of time. The fluxes were obtained from the linear portion of the accumulation curve, considering the slopes of the regression line (angular coefficient). Diffusion coefficients were calculated according to Equation (6).

*D* = *F*/[CA] (6)

where *D* is the diffusion coefficient and *F* is the flux and [CA] is the CA concentration in the analyzed form, expressed in mg/mL.

### 2.14. HPLC Procedure

For HPLC analyses, a two-plungers alternative pump (Agilent Technologies 1200 series, Santa Clara, CA, USA), an UV-detector operating at 325 nm, and a 7125 Rheodyne injection valve with a 50 μL loop were employed. Analyses were conducted eluting a stainless-steel C-18 reverse-phase column (15 × 0.46 cm) packed with 5 μm particles (Platinum C18, Apex Scientific, Alltech, Nicholasville, KY, USA) with a mobile phase containing acetonitrile/water 20:80 *v*/*v*, pH 2.5 at a flow rate of 0.7 mL/min. In those conditions the CA retention time was 4.5 min [37].

### 2.15. In Vitro Assessment of CA-Hydrogen Peroxide Reactions in Skin Using SCOE

Amperometric measurements were conducted to detect the CA penetration through the skin. The mechanism of the SCOE is illustrated in supplementary material (Figure S1). The preparation of pig ear skin membranes and assembly of SCOE were obtained as reported previously [26]. Video of skin membrane preparation can be found at [41]. The ethical permission to use the skin from pig ears is not required in this case, since the ears are rest material from pigs slotted for food preparation. The assembling of SCOE, in brief was done as follows. Oxygen electrode was covered with 500 µm thick and 16 mm in diameter excised porcine skin membrane and fixed by rubber o-ring. Amperometric measurements with SCOE were performed with the electrode immersed into electrochemical measurement cell (glass) filled with 10 mL of citrate buffer saline (CBS) constituted of 10 mM sodium citrate dihydrate, citric acid monohydrate, and 150 mM sodium chloride, pH 5.5, under continuous magnetic stirring. The current of the electrode was recorded using AMEL model 2059 potentiostat/galvanostat (AMEL, Milano, Italy) by applying −0.7 V (vs Ag/AgCl) on the Pt electrode of the oxygen electrode. When baseline was stable, the response to 0.5 mM H_2_O_2_ was recorded. After the stabilization of the response to H_2_O_2_, CA (0.5 mM) in ethanol/CBS (7:3 *v*/*v*) solution (CA-Sol), ETHOs or CA-ETHOs were added into the measurement cell. All measurements have been conducted at 32 °C.

## 3. Results and Discussion

### 3.1. Caffeic Acid Solubility and Stability

**Figure 1:**
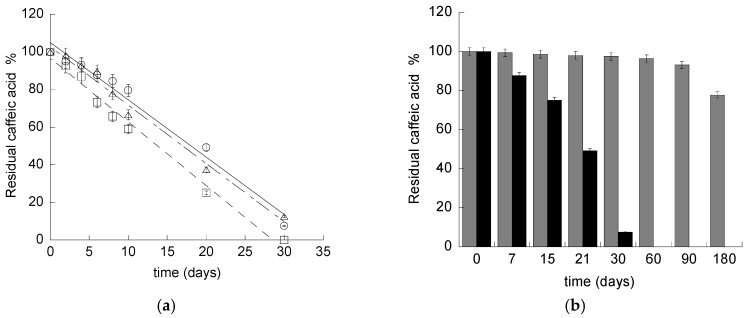
(**a**) Caffeic acid content in aqueous solution stored at 22 (open circle), 4 (triangle), or 40 (square) °C for 30 days. (**b**) Caffeic acid content in aqueous solution (black) or in ethosome (ETHO) (grey) stored at 22 °C.

In order to determine the CA stability in water, its content was evaluated in aqueous solution of the drug stored in different conditions. Mass spectrometry analyses revealed that CA was soluble in water up to 0.5 mg/mL. CA in aqueous solution underwent complete degradation within one month (Figure 1a). As expected, the degradation was influenced by the temperature, being more rapid in the case of the CA solution stored at 40 °C with respect to 22 and 4 °C.

The mathematical modelling of CA residual content, obtained by fitting profiles with Equations (1) and (2), revealed that CA stability followed a zero order kinetic model, as indicated by the high coefficient correlation values reported in Table S1, suggesting that CA stability was independent from its concentration [29].

In order to control CA stability, the possibility to entrap the drug within a nanotechnological formulation was investigated.

### 3.2. Preparation of Ethosomes

Since CA is soluble in ethanol (up to 50 mg/mL [42]), ETHOs were selected for CA delivery, being biocompatible transdermal nano-systems, containing high amounts of ethanol. Particularly, ETHO were constituted of PC ethanol solution and water (30:70 *v*/*v*), as reported in Table 1. ETHOs were spontaneously and rapidly obtained by a cold method, dropping water into a PC ethanol solution under magnetic stirring, finally leading to milky dispersions. Water addition occurred within 20 s, afterwards the vials have been sealed, preventing ethanol evaporation, indeed ethanol concentration, determined by gas chromatography with thermal conductivity detection [30] was actually 30 ± 1% *v*/*v* (*n* = 3). CA-ETHOs were obtained after CA solubilization in the PC ethanol solution, followed by addition of water. ETHO and CA-ETHO compositions are reported in Table 1.

The *EE* of CA in ETHO, evaluated by ultracentrifugation and HPLC analysis, indicated that CA was completely associated to vesicles (*EE* = 98 ± 2 %). The high EE achieved by CA-ETHO should be attributed both to the presence of ethanol and to the simple production method, avoiding high energy input, such as heating and mechanical stresses, preserving CA from possible degradation [14,22]. In this respect CA encapsulation in ETHO appears more advantageous with respect to another PC based nanovesicle system recently described by Permana et al. [29]. Indeed, the authors reported a more complex preparation procedure, based on rotary evaporation followed by homogenization and sonication, leading to a 45% *EE* of CA at the latest [29].

The capability of ETHO to solubilize CA has been further investigated, revealing that the upper limit was 5 mg/mL, thus 10-fold higher with respect to CA solubility in water. This result is ascribable to the CA solubility value in ethanol/water (30:70 *v*/*v*), being 5.5 mg/mL.

### 3.3. Characterization of Ethosomes

**Figure 2:**
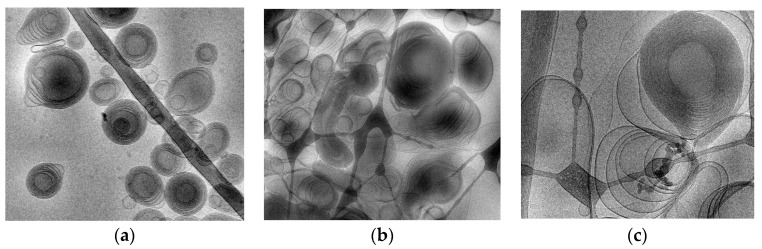
Cryo-transmission electron microscopy images (Cryo-TEM) of CA-ETHO (**a**) and CA- ETHO-pol (**b**,**c**). Bar corresponds to 200 nm (**a**,**b**) or 100 nm (**c**).

To shed light on the structure and dimensions of ETHO, cryo-TEM, PCS and SAXS have been employed. Figure 2a reports a cryo-TEM image of CA-ETHO, showing the typical fingerprint structure, ascribable to the double layer organization of PC, resulting in multilamellar vesicles with spherical, as well as elongated shape.

Regarding size distribution, mean diameters expressed as Z Average were around 200 nm, with very slight differences between ETHO and CA-ETHO. PCS analyses evidenced a monomodal size distribution, as indicated by dispersity index values below 0.2 (Table 2).

**Figure 3:**
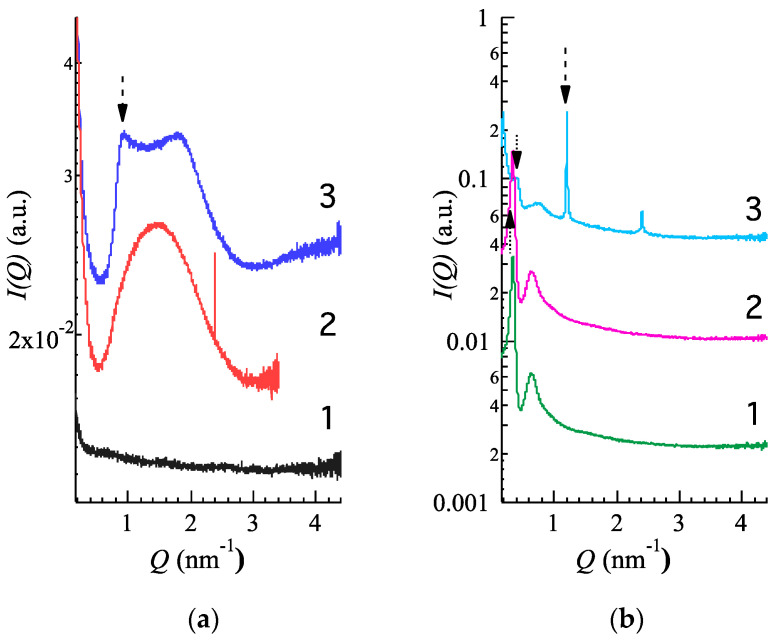
SAXS profiles observed at 35 °C. (**a**): water (1, black), ETHO (2, red), CA-ETHO (3, blue); (**b**): pol (1, green), CA-pol (2, violet), CA-ETHO-pol (3, light blue). Arrows indicate the main diffraction peaks.

SAXS experiments were performed to confirm the structural properties of ETHO. Figure 3a reports water (1), ETHO (2) and CA-ETHO (3) scattering profiles. In the case of ETHO, a typical bilayer form factor scattering pattern (e.g., the broad band centered at about 1.5 nm^−1^) is observed. Such a profile denotes the presence of PC unilamellar vesicles or, more probably, PC multilamellar vesicles characterized by a very disordered positional correlation between adjacent bilayers and/or by a very limited number of stacked bilayers [22]. On the other hand, the X-ray scattering pattern of CA-ETHO is characterized by low-intensity Bragg peaks superposed to the bilayer form factor as a clear indication of the formation of PC multilamellar vesicles. Therefore, CA presence appears to induce a small but clear increment of the positional correlations between adjacent bilayers [43]. The bilayer-to-bilayer repeat distance, derived from the peak positions and corresponding to the sum of the bilayer thickness plus the thickness of the water layer separating two adjacent bilayers, was 6.90 nm. It should be noted that other authors have found that the incorporation of hydrophobic phenolics into lipid vesicular systems bilayers could thoroughly affect the bilayer organization [44].

### 3.4. Evaluation of CA Antioxidant Activity

The antioxidant activity of the CA-ETHO was evaluated using the DPPH radical scavenging activity and photochemiluminescence methods [34,35,36]. Both methods demonstrated that CA encapsulation within ETHO did not affect its antioxidant capacity. Indeed, IC50 values measured by DPPH were 10 µg/mL and 7 µg/mL, respectively, for CA-ETHO and CA in ethanol solution, while photochemiluminescence revealed an antioxidant capacity of 49.0 ± 0.9 and 46.4 ± 0.7 μmol TE/mL for CA-ETHO and CA ethanol solution respectively

### 3.5. Evaluation of CA-ETHO Stability

In order to check the size stability of ETHO, size distribution of samples stored in the light at 22 °C was evaluated by PCS periodically for three months. As reported in Figure 2a and Table 2, vesicle size remained quite stable, indeed mean diameter slightly increased (≈20 nm) after 90 days from ETHO and CA-ETHO production.

CA content in ETHO has been evaluated within six months from CA-ETHO preparation, to detect the effectiveness of ETHO in controlling CA degradation. Noteworthily, some authors have investigated the possibility to entrap CA in vesicular or nanoparticulate system, however, to the best of our knowledge, the capability to control CA degradation has not been yet underlined [29,45,46,47,48]. Remarkably, after three months, CA content in ETHO was almost unvaried, being 93% of the initial content, while, after six months, 77% of CA was still present in CA-ETHO (Figure 1b). Conversely, a CA ethanol/water solution (30/70, *v*/*v*) taken as control, stored at 22 °C, underwent a CA 15% loss after just three days, confirming the role of ETHO in maintaining CA stability.

### 3.6. Ethosome Gel Preparation and Characterization

ETHO dispersions need to be thickened to be applied on the skin [49,50]. At this purpose, the copolymer p407 was directly added to the ETHO dispersions under stirring, up to a final 15% *w*/*w* concentration. The addition of p407 slightly diluted both ETHO and CA-ETHO (Table 1). However, ETHO-pol and CA-ETHO-pol maintained the milky homogeneous aspect of ETHO, while gaining a semi-solid consistency. It is well known that p407 in water self-aggregates, giving rise to spherical polymeric micelles with a PPO hydrophobic core and a hydrophilic PEO screen [22,37]. Upon the addition of p407 into ETHO dispersions, reasonably, an interaction occurred between the polymer and the multilamellar PC structure constituting the membrane of ETHO vesicles. Moreover, the formation of polymeric micelles, as well as mixed PC-p407 micelles, is conceivable.

In order to detect the influence of p407 on the architecture of CA-ETHO dispersions, their morphology was visualized by cryo-TEM. Figure 2b,c refer to CA-ETHO-pol, showing irregularly shaped oligo lamellar vesicles with multiple lipid sheets. Particularly, in Figure 2c, a big multilamellar fingerprint vesicle is detectable, as in the case of CA-ETHO. Cryo-TEM analysis suggests that, even if the addition of p407 affected the regular spherical shape of CA-ETHO, the typical supramolecular organization of PC was preserved.

Further insights on the structure of thickened vesicles, especially on the ETHO structural changes due to p407 addition, have been obtained by SAXS. Namely CA-ETHO-pol has been compared to p407 gel (pol) and to p407 gel loaded with CA (CA-pol), whose compositions are reported in Table 1. SAXS results are reported in Figure 3b, showing very similar curves in the case of pol (1) and CA-pol (2), being characterized by two low angle peaks, indicating that no effects were induced by CA addition on p407 aggregational properties. Notably, recent model fitting has demonstrated that the two peaks are related to the disordered organization of the polymer micelles in a 3D cubic structure, with a lattice constant of about 20 nm [37]. In this respect CA-pol profile indicates that 3D organization was not altered by the presence of CA. Conversely, in the case of CA-ETHO-pol (Figure 3b), the two low angle peaks are further detectable, although in a shifted position, together with strong and narrow Bragg peaks, evidencing that the addition of p407 in CA-ETHO led to a very ordered multilamellar structure. Consequently, the following three points should be stressed:

(i) The diffraction peaks of CA-ETHO-pol were narrower and more intense with respect to CA-ETHO (Figure 3a, profile 3), suggesting that the diffuse background between the Bragg peaks was no longer modulated by the bilayer form factor. Therefore, the addition of p407 strongly increased the positional correlations between adjacent PC bilayers.
(ii) The repeat distance for CA-ETHO-pol appeared 5.20 nm, that is 1.7 nm minor than CA-ETHO, suggesting a diminished hydration of the bilayers.
(iii) The 3D disordered cubic packing observed in CA-pol was maintained in the presence of ETHO, though the lattice constant was ≈15 nm, hence decreased of roughly 5 nm.

**Figure 4:**
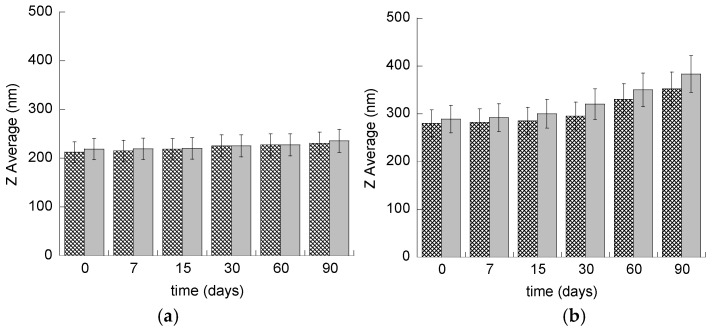
Mean diameter variation for ETHO (**a**) and ETHO-POL (**b**) unloaded (crisscross pattern) or CA loaded (grey pattern). Diameters have been measured by PCS during time, up to three months from production and expressed as Z average. Data are the mean of three determinations on different samples.

Regarding size distribution, PCS data reported in Table 2 evidenced that mean diameters of ETHO and CA-ETHO underwent roughly a 70 nm increase under p407 thickening, but still maintained a narrow size distribution, as indicated by dispersity indexes. As reported in Figure 4b and Table 2, Z Average mean diameters of ETHO-pol and CA-ETHO-pol were almost stable up to 30 days, afterwards they sustained respectively an 80 nm and 100 nm increase. Three months from CA-ETHO-pol preparation, the Z average mean diameter was 383 nm, and the polydispersity index was 0.26, indicating that the vesicle size distribution was not particularly affected by aging.

### 3.7. Rheological Study

**Figure 5:**
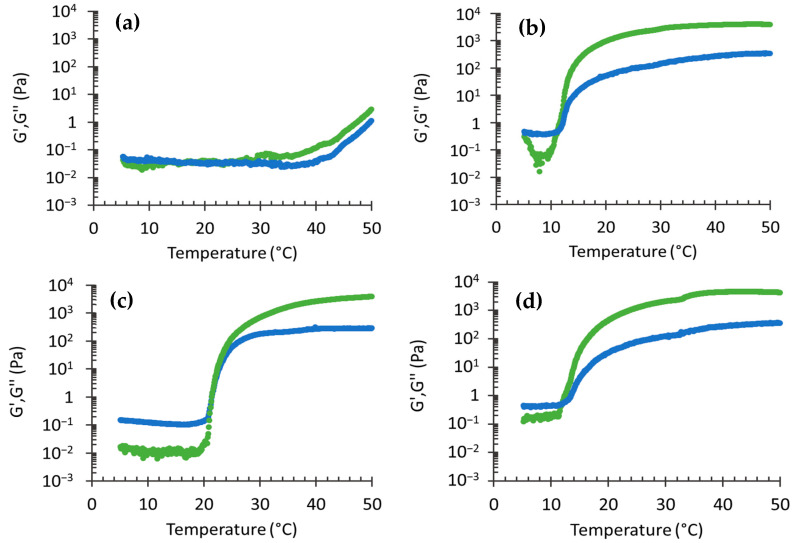
Effect of the temperature on storage (G’, green) and loss (G’’, blue) moduli for ETHO (**a**), ETHO-pol (**b**), CA-pol (**c**), and CA-ETHO-pol (**d**).

In order to straighten out the impact of p407 presence on CA-ETHO and to have information on the thermal response of ETHO formulations, their rheological behavior has been studied. Particularly the viscoelastic properties have been investigated, evaluating the storage G’ and loss G’’ moduli. The storage modulus represents the energy stored in the elastic structure of the formulation, while the loss modulus reflects the viscous part or the dissipated energy [51]. Figure 5 reports the G’ and G’’ profiles for ETHO, ETHO-pol, CA-pol, and CA-ETHO-pol. In the case of the liquid vehicle ETHO, G’ and G’’ profiles were roughly constant with low values and overlapped up to 35 °C; afterwards they slightly increased, up to 50 °C. On the other hand, in the case of thickened forms, the thermal behavior was very different. Indeed, profiles were characterized by an initial phase where G’’ overcame G’ at low temperature, followed by a second phase with an inverse trend, with G’ higher than G’’ at higher temperature. This rheological behavior is characteristic of the thermogelling properties of p407. The inflection points correspond to the *T*_sol-gel_ temperature, indicating the transition from liquid to structured gel [52]. Since the storage modulus profile overcame the loss modulus, ETHO-pol, CA-pol and CA-ETHO-pol can be considered as mainly elastic above *T*_sol-gel_. It is interesting to note that the *T*_sol-gel_ in the case of CA-pol was roughly 22 °C (Table 3), while the presence of ETHO substantially reduced *T*_sol-gel_ by 10 °C. Indeed, the presence of vesicles in ETHO-pol creates a more structured gel network with respect to the pol gel. Moreover, CA presence slightly increased the sol-gel transition temperature.

### 3.8. Deformability Study

One of the peculiar features of ETHO is related to their deformability, enabling them to pass intact through natural membranes, such as the skin [53]. To study the effect of CA and p407 on the deformability of ETHO, the variation of mean diameter has been evaluated under vesicle extrusion. The extrusion was conducted at 25 °C since, at higher temperatures, CA-ETHO-pol viscosity prevented their passage through the extruder. The results are summarized in Table 3. CA-ETHO were rather more elastic than unloaded ETHO. Notably, ETHO and CA-ETHO were characterized by deformability values 10- and 20-fold higher with respect to the thickened forms. Thus, the presence of p407 decreased the vesicle deformability, indeed as demonstrated by SAXS measurements, in the case of CA-ETHO-pol, the polymer promotes the formation of packed multilamellar structures.

### 3.9. In Vitro CA Diffusion Kinetics

**Figure 6:**
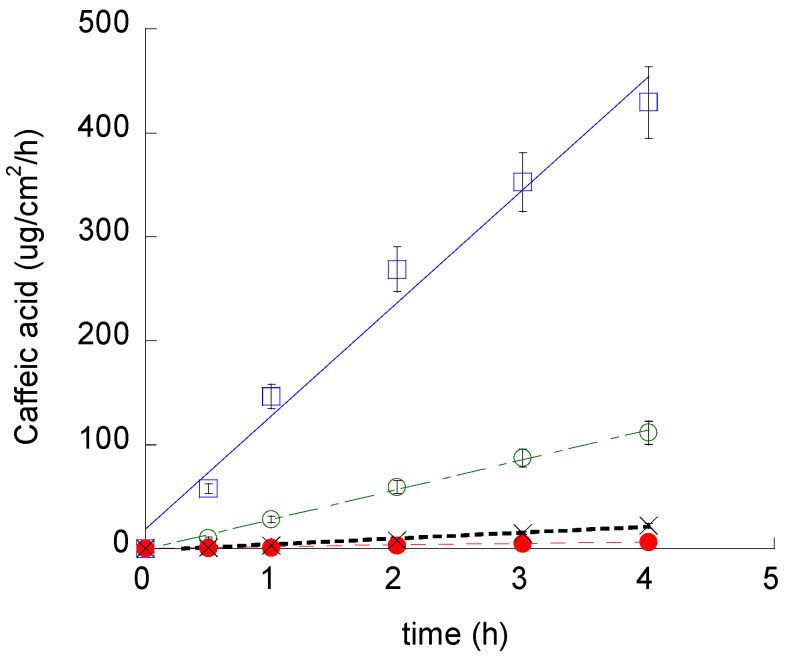
CA diffusion kinetics from CA-water (blue square), CA-pol (green open circle), CA-ETHO (black crosses), and CA-ETHO-pol (red closed circle), as determined by Franz cell. Data are the mean of six independent experiments.

With the aim of gaining information on the efficiency of ETHO as cutaneous vehicles for CA, its diffusion kinetics were studied in vitro by Franz cell associated to nylon membranes [36]. We chose to employ a synthetic membrane to perform an initial comparative screening between the different formulations, while porcine skin membrane was further employed for ex-vivo evaluation. Namely, CA-ETHO-pol was investigated and compared to CA-pol and the aqueous solution CA-water (Figure 6). From the slopes of the linear profiles, F values were obtained, and D values were calculated (Equation (6)) and reported in Table 4. As expected, the fastest diffusion was obtained in the case of CA-water, followed by CA-pol, from which CA diffusion was almost four-fold slower. CA diffusion from CA-ETHO was dramatically slower; indeed, D values were 137-fold lower with respect to CA-water, suggesting that the multilamellar organization of the vesicles firmly held CA and controlled its diffusion. Other authors described multilamellar vesicles as more suitable to control drug release with respect to unilamellar ones [54]. The vesicle role in controlling CA diffusion was also confirmed in the case of the thickened vehicle CA-ETHO-pol; indeed, the drug diffusion was 8.6-fold lower with respect to CA-pol. On the other hand, CA diffusion from CA-ETHO was four-fold slower than from CA-ETHO-pol. This behavior was unpredictable; indeed, the viscous structure of the ETHO gel was expected to restrain CA diffusion. It can be hypothesized that, in the case of CA-ETHO-pol, the addition of p407 affected the strong CA association to the PC bilayers, as indicated by the diminished hydration of the bilayers and increased order found by SAXS (Figure 3b). Thus, CA could be more tightly associated to the PC bilayer of ETHO vesicles with respect to ETHO-pol structure, from which CA can diffuse faster.

### 3.10. Ex-Vivo Evaluation of Permeation and Role of CA in Antioxidant Reactions in Skin

Amperometric measurements were conducted to detect the CA permeation through the skin. Particularly, SCOE was prepared by placing skin membrane on the tip of oxygen electrode. The electrode allows registration of O_2_ concentration changes in the membrane due to reactions of H_2_O_2_ and polyphenols in the skin. In brief, when SCOE is immersed into buffer solution, the electrode current (baseline current) corresponds to 0.26 mM of dissolved O_2_ in the solution. To model inflammatory condition with elevated reactive oxygen species (ROS) in skin, H_2_O_2_ is added into the solution. The H_2_O_2_ permeates through the *stratum corneum* and gives rise to additional O_2_ (Equation (7)), thus increasing electrode current, due to catalase enzyme abundantly present in the epidermis:

(7) $$2H_{2}O_{2}\overset{Cat}{\to }2H_{2}O+O_{2}$$

The possible polyphenol penetration and its engagement in the antioxidative reactions are indicated by the SCOE, showing the reduction of the electrode current. To understand this amperometric response, it should be considered that a number of antioxidative enzymes in skin possess peroxidase type activity (Equation (8)), which, in the presence of polyphenols, consume H_2_O_2_ (Equation (8)), leaving less of it for the catalase reaction (Equation (7)). The peroxidase type reaction, in case of the polyphenol CA as a hydrogen donor, for reduction of H_2_O_2_ to water is specified below.

(8) $$2H_{2}O_{2}+CA\overset{Peroxidase\;like\;enzymes\;in\;skin}{\to }2H_{2}O+CA_{ox}$$

**Figure 7:**
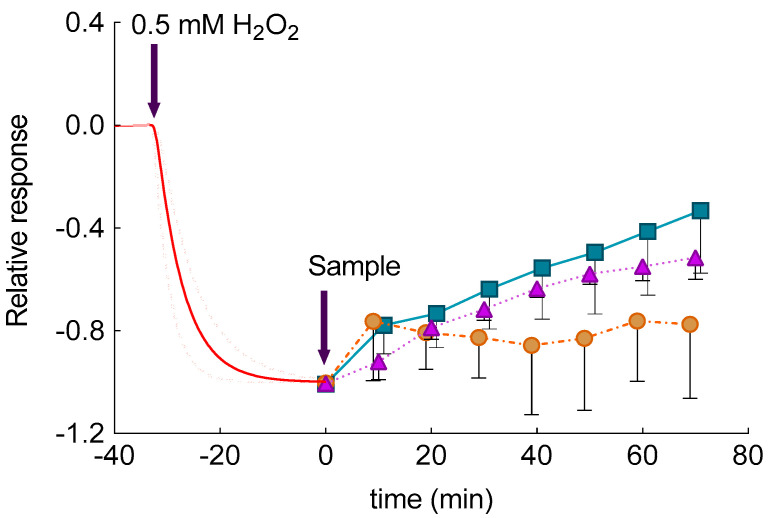
Relative, average amperometric current response of skin covered oxygen electrode immersed in CBS, pH 5.5, to addition of H_2_O_2_ (0.5 mM, red dotted and solid lines, *n* = 13) CA sol (CA 0.5 mM, triangle, *n* = 3); CA-ETHO (CA 0.5 mM, square, *n* = 6) and unloaded ETHO (circles, *n* = 4).

In a previous study, the possibility of SCOE to monitor the kinetics of polyphenol penetration through *stratum corneum* and its engagement in antioxidative reactions in the presence of H_2_O_2_ in skin has been demonstrated [25]. SCOE allowed us to assess permeability and engagement of CA in antioxidant reactions, which are catalyzed by peroxidase like enzymes present in skin. The results of the measurement with this electrode are presented in Figure 7 and can be explained by the following. After the SCOE is immersed in CBS, approximately 30 min are needed to get steady-state current, indicating that skin, O_2_ distribution in the skin as well as O_2_ diffusion profiles in the skin have equilibrated after immersion of SCOE into CBS. As can be seen from Figure 7, when 0.5 mM of H_2_O_2_ is injected into the measurement cell filled with CBS, a gradual increase in reduction current (reduction current is regarded as negative by definition) has been observed, due to H_2_O_2_ penetration into the skin and production of O_2_ by catalase reaction (Equation (7)). Due to biological variability of skin, the SCOE response to H_2_O_2_ is presented as an average response (Figure 7). As can be seen from Figure 7, a steady-state current is reached after approximately 30 min, indicating that equilibrium between H_2_O_2_ penetration/diffusion in skin and catalase driven O_2_ production has been reached. This current response is considered as 100% response to H_2_O_2_. Since H_2_O_2_ is, currently, present in the skin, the experiment setup models inflammatory condition in skin with respect to elevated ROS. The time moment at which this model situation is reached is considered as “time zero” for studies of polyphenols antioxidant effect in the skin. At this time, the CA was introduced into the measurement cell in form of CA solution (CA-Sol) or CA-ETHO. Upon addition of CA-ETHO and CA-sol, the antioxidant effect of CA has been revealed by SCOE response to lower current (Figure 7), indicating that an amount of H_2_O_2_ was reduced to water by CA, due to a peroxidase-like reaction in the skin (Equation (8)). Noticeably, unloaded ETHO introduced into the cell at time zero exerted an initial response within 10 min. Afterward, unloaded ETHO did not produce a significant change, returning to base line, whereas CA-ETHO kept increasing the SCOE response in the following 60 min. The initial response could be probably ascribed to the antioxidant and barrier enforcing properties of PC [55,56], conferring antioxidant activity also to unloaded ETHO. On the other hand, in the case of ETHO loaded with an antioxidant molecule such as CA, a synergistic effect was exploited. In the case of CA-sol, SCOE response was appreciable, although more delayed and less intense with respect to CA-ETHO. These results suggested that both CA-sol and CA-ETHO were engaged in reduction of H_2_O_2_. The antioxidant effect exerted by CA-ETHO as well as CA penetration were more intense with respect to the drug in solution, demonstrating that CA delivery through the skin should be ascribed to the ETHO supramolecular structure and not merely to the ethanol presence. The integrity of skin membranes was assessed by electrical impedance measurements of their resistance [25]. The measured values were higher than 3 kΩ, demonstrating that skin membranes have good integrity, i.e., intact *stratum corneum*.

SCOE findings agree well with those obtained by diffusion experiments in terms of effective delivery of CA through membranes. CA diffusion through synthetic nylon membrane was retarded by ETHO (lower apparent diffusion coefficient, Table 3), while experiments with SCOE clearly showed that ETHO formulation promoted CA delivery into skin. Thus, the present data corroborated previous findings indicating the transdermal potential of ETHO [15,16,17,18].

## 4. Conclusions

The results of this study demonstrated that ETHO can be successfully employed to encapsulate CA with higher EE, with respect to other nanosystems previously described, and to control CA chemical stability, long maintaining its antioxidant potential. Experiments with skin-covered oxygen electrodes suggested that ETHO enhanced delivery of CA and its antioxidant action to porcine skin with respect to CA delivery from simple solution. In this respect, it can be asserted that the transdermal effect has to be ascribed to ETHO and not to the presence of ethanol alone, which could act as penetration enhancer. Nonetheless, future studies will be undertaken in order to better investigate the interaction of ETHO with skin and/or mechanism of CA delivery by ETHO.

## Figures and Tables

**Table 1:** Composition of the indicated formulations.

Formulation Code	PC ^1^ % *w*/*w*	Ethanol % *w*/*w*	P407 ^2^ % *w*/*w*	Water % *w*/*w*	CA ^3^ % *w*/*w*
ETHO	0.9	29.1	-	70	-
CA-ETHO	0.9	29.1	-	70	0.1
pol ^4^	-	-	15	85	-
CA-pol ^5^	-	-	15	84.95	0.05
ETHO-pol ^6^	0.76	24.74	15	59.5	-
CA-ETHO-pol ^7^	0.76	24.74	15	59.41	0.085

^1^ soy phosphatidylcholine; ^2^ poloxamer 407; ^3^ caffeic acid; ^4^ p407 gel; ^5^ CA containing p407 gel; ^6^ ethosome gel; ^7^ CA containing ethosome gel.

**Table 2:** Dimensional distribution parameters of ethosomes and ethosome gels, as determined by photon correlation spectroscopy (PCS).

Formulation Code	Time (Days)	Z Average (nm) ± s.d.	Dispersity Index ±s.d.
ETHO	0	212.25 ± 14.10	0.12 ± 0.01
	90	230.52 ± 25.30	0.14 ± 0.03
CA-ETHO	0	218.65 ± 21.0	0.20 ± 0.02
	90	235.45 ± 20.01	0.22 ± 0.03
ETHO-pol	0	279.9 ± 24.30	0.16 ± 0.02
	90	352.15 ± 42.01	0.23 ± 0.03
CA-ETHO-pol	0	288.8 ± 35.50	0.24 ± 0.01
	90	383.25 ± 47.12	0.26 ± 0.03

s.d.: standard deviation; data are the mean of 3 independent determinations on different batches.

**Table 3:** Deformability parameters and transition temperatures of the indicated formulations.

Formulation Code	Vesicle Size ^1^ (nm)	Def ^2^ (mL/min*)*	*T*_sol-gel_ (°C)
ETHO	109.13 ± 1.07	6.21 ± 0.52	-
CA-ETHO	116.80 ± 1.60	8.06 ± 0.46	-
CA-pol	-	-	21.87 ± 0.99
ETHO-pol	186.93 ± 17.66	0.61 ± 0.04	11.43 ± 0.47
CA-ETHO-pol	194.13 ± 9.71	0.40 ± 0.16	13.60 ± 2.13

^1^ Z Average, as measured by PCS after extrusion; ^2^ vesicle deformability; data are the mean of three independent determinations on different batches.

**Table 4:** Fluxes and diffusion coefficients of the indicated formulations.

Formulation Code	F ^1^ ± s.d. (mg/cm^2^/h)	CA (mg/mL)	D ^2^ ± s.d. (cm/h) × 10^−3^
CA-water	108.77 ± 13.4	0.5	217.54 ± 26.8
CA-pol	28.70 ± 3.4	0.5	57.40 ± 6.8
CA-ETHO	1.59 ± 1.1	1	1.59 ± 1.1
CA-ETHO-pol	5.64 ± 2.4	0.85	6.63 ± 2.82

^1^ Flux; ^2^ Diffusion coefficient; data are the mean of six independent Franz cell experiments.

## Supplementary Materials

The following are available online at https://www.mdpi.com/1999-4923/12/8/740/s1. Table S1: Kinetic modelling of stability profile of CA aqueous solution stored for 30 days at different temperatures, Figure S1: Schematic representation of mechanism of skin covered oxygen electrode.
