# Supplementary material for: Design and Characterization of Ethosomes for Transdermal Delivery of Caffeic Acid
Source: Pharmaceutics. 2020 Aug 6;12(8):740. doi: 10.3390/pharmaceutics12080740 (PMC7465088; doi:10.3390/pharmaceutics12080740)
Supplement: Supplementary file 1 [file pharmaceutics-12-00740-s001.pdf]

pharmaceutics

Supplementary Materials: Design and Characterization of Ethosomes for Transdermal Delivery of Caffeic Acid

Supandeep Singh Hallan, Maddalena Sguizzato, Paolo Mariani, Rita Cortesi, Nicolas Huang, Fanny Simelière, Nicola Marchetti, Markus Drechsler, Tautgirdas Ruzgas and Elisabetta Esposito

Table S1. Kinetic modelling of stability profile of CA aqueous solution stored for 30 days at different temperatures.

Kinetic Parameters	4 °C<br>*R²/K*	22 °C<br>*R²/K*	40 °C<br>*R²/K*
Zero order	0.991/3.442	0.968/3.256	0.986/3.382
First order	0.760/0.197	0.716/0.207	0.869/0.142

data are the mean of 3 independent experiments, S.D. were within ± 5%

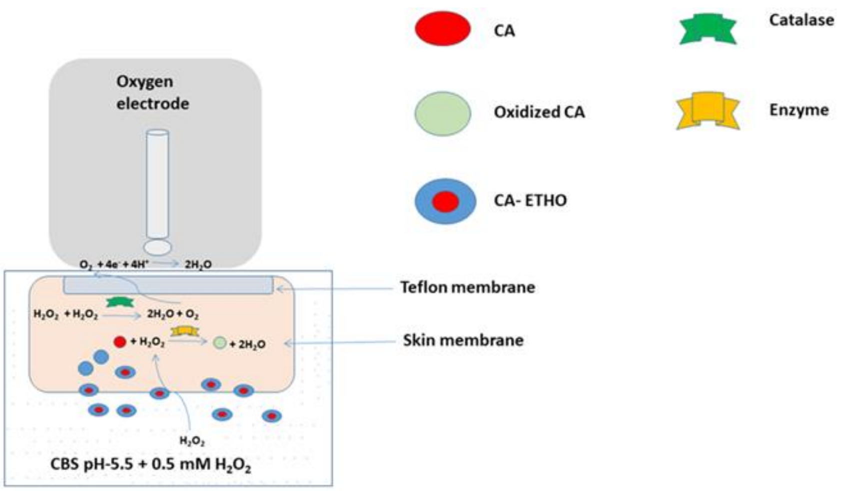

Figure S1. Schematic representation of mechanism of skin covered oxygen electrode. The equations 4 and 5 in the main text are illustrated here by reactions involving catalase and enzyme (with peroxidase like activity). Oxygen electrode current is proportional to O_2 concentration in skin, which is modulated by the extent of both mentioned reactions, since they compete for H_2O_2 as a common substrate. Catalase reaction produce O_2 from H_2O_2 (increases SCOE current), while peroxidase like activity in skin generates water from H_2O_2 (decreases SCOE current). Analyzing current responses to polyphenols in the presence of H_2O_2 allows to assess apparent kinetics of transdermal/topical delivery of antioxidant property of the polyphenol into skin.
